# Predictive modelling of the dynamics of antimicrobial resistance: creation of a bank of renewable models based on machine learning

**DOI:** 10.3389/fphar.2026.1715346

**Published:** 2026-02-09

**Authors:** M. A. Arepyeva, A. Y. Kuzmenkov, A. A. Starostenkov, A. S. Kolbin, Y. E. Balykina, Yu M. Gomon, A. A. Kurylev, R. S. Kozlov, S. V. Sidorenko

**Affiliations:** 1 Saint-Petersburg State University, Saint-Petersburg, Russia; 2 Institute of Antimicrobial Chemotherapy, Smolensk State Medical University, Smolensk, Russia; 3 Pavlov First Saint-Petersburg State Medical University, Saint-Petersburg, Russia; 4 North-Western State Medical University Named After I. I. Mechnikov, Saint-Petersburg, Russia

**Keywords:** antimicrobial resistance prediction, antimicrobials, consumption, defined daily dose, machine learning, pharmacoepidemiology

## Abstract

**Introduction:**

The growth in antimicrobial resistance (AMR) presents a global threat, caused to a large extent by irrational antimicrobial consumption. For its part, mathematical modelling of the dynamics of AMR on the basis of data on antimicrobial consumption and historical levels of resistance may prove a promising tool for optimizing strategies to control this problem.

**Methods:**

We used data on the consumption of systemic antimicrobials in the period 2008-22 for 82 regions of the Russian Federation and AMR levels in the period 2013-22. The data was processed with standardization of the regional names, the exclusion of antimicrobials with insignificant usage, the calculation of moving averages for AMR (with a window of 3-10 years) and antimicrobial consumption in Defined Daily Doses. To reduce dimensionality, principal component analysis was employed. On the basis of the “model pair” (microorganism-antibiotic) concept we tested machine learning algorithms: Light Gradient Boosting Machine (LightGBM), Random Forest, logistic regression, Support Vector Machines (SVMs) with linear and Gaussian kernels. We performed the calibration of the hyperparameters with cross-validation and assessed the metrics of precision and recall. We carried out predictions of AMR for optimized Constrained Optimization BY Linear Approximation (COBYLA method) and realistic Error, Trend, Seasonality (ETS model) usage scenarios.

**Results:**

For the model pair *E. coli*–cefotaxime, the LightGBM model presented the highest precision (67.5% for the training set, 66.6% for the validation set) without indications of overfitting. The key predictors of AMR would be the moving average of historical resistance and the type of infection. Optimization of consumption structure by the COBYLA method made it possible to predict a 15–20% reduction in AMR over 10 years, while a realistic scenario ETS foresaw a 5–10% growth in resistance.

**Discussion:**

The bank of models created on the basis of LightGBM provides for precise forecasting of the dynamics of AMR and the formation of strategies for the management of antimicrobial therapy. An optimization of consumption according to the results of the modelling is capable of reducing resistance by 15–20%. The AMCmodel.ru platform provides tools for real-time decision-making. An online platform AMCmodel.ru has been developed for data visualization, access to models and forecast generation.

## Introduction

In a 2024 report, World Health Organization (WHO) experts predicted that by 2050 resistance to antimicrobials will become the direct cause of 1.91 million deaths worldwide and be associated with a further 8.22 million (Naghavi et al., 2024). This is an indication that without additional measures, the 10% reduction in mortality from antimicrobial resistance (AMR) previously indicated in WHO programmatic declarations (The Lancet, 2024) will not be achieved. In 2024 the WHO named as critical-priority pathogens carbapenem-resistant *A. baumannii*, carbapenem-resistant Enterobacterales, Enterobacterales resistant to third-generation cephalosporins and rifampicin-resistant *M. tuberculosis*; while methicillin-resistant *Staphylococcus aureus* (MRSA) was assigned to the category of high-pathogens (World Health Organization, 2024). The most discussed cause of growth in AMR is stated to be improper use of antimicrobials (antimicrobial consumption–AMC) within the healthcare system (Holmes et al., 2016; World Health Organization, 2021). In order to test this hypothesis pharmaco-epidemiological studies need to be carried out, such as the qualitative and quantitative Drug Utilization Review (DUR) (World Health Organization, 2015).

In order to develop qualitative indicators of AMC, and also to determine target figures, in 2017 the WHO produced the AWaRe (Access, Watch, Reserve) classification (World Health Organization, 2022).

The classification calls for antimicrobials to be divided into three categories: “General Availability” (Access), “Limited Availability” (Watch) and “Reserved” (Reserve). The WHO set a target calling for no less than 60% of antibiotic consumption at a national level to belong to the Access category (Sulis et al., 2022; World Health Organization, 2022). The reduction of incidences of inappropriate usage of antibiotics in the Watch and Reserve categories is one of the important components of the struggle against AMR.

Quantitative analysis for the study of antimicrobial consumption is performed using the technical unit ATC/DDD (Defined Daily Dose (DDD). Definition and general considerations., 2025). Most often DDD is used in the study of changes in the use of medicines over time, in comparative studies between regions and countries, in the assessment of the influence of administrative instructions and recommendations on the consumption of medicines and in documenting different groups’ relative intensity of usage.

Antimicrobial stewardship programmes propose a whole range of measures to reduce AMR: working cooperation between microbiologists and clinical pharmacologists, improvements in the equipment of laboratory complexes in medical organizations, the development of guidelines for the treatment of infections on the basis of local microbiological data, educational programmes for doctors, the implementation of targeted epidemiological surveillance, studies of the quantitative and qualitative usage of antimicrobials (Drug Utilization Review, DUR) and so on. (Fishman, 2006).

One of the modern approaches to the management of AMR is considered to be the use of digital healthcare, pharmacometrics, machine learning and modelling (The Lancet Digital Health, 2024). The construction of mathematical forecasting models is increasingly being used to obtain an understanding of the pathogenesis of infectious diseases and the struggle against them, among other things to identify key parameters determining the spread of diseases, the assessment of the effect of potential interventions and forecasting the development trajectory of epidemics (Heesterbeek et al., 2015). Over the period 2006–16, the pathogens responsible for five infectious predominated in mathematical models for forecasting AMR: methicillin-resistant *S. aureus* (MRSA), tuberculosis, the human immunodeficiency virus (HIV), influenza and malaria. The models took into account the influence of various regimens for the consumption of antibiotics, observance of the rules of asepsis and antisepsis, and infection control, as well as screening and diagnostics. Less than 5% of the models included the use of alternative therapeutic strategies or changes in AMC strategies (Niewiadomska et al., 2019).

The regression model for predicting AMR previously developed in Russia was based on data about the quantitative consumption of antimicrobials and the dynamics of bacterial resistance in *E. coli* to third-generation cephalosporins and inhibitor-protected penicillins. In the course of data-collection for the modelling, an analysis was made of the Saint Petersburg’s WHONET database for the period 2007–12, and then a cross-sectional retrospective clinical study was carried out based on one of the city’s medical institutions (Arepyeva et al., 2017). The model showed that quantitative analysis of antimicrobial consumption and AMR data can be used as a basis for making short-term and medium-term forecasts for resistance over the next 6–12 months. Parametric and non-parametric approaches were assessed, linear and non-linear models were tested with regard to the introduction of antimicrobial consumption (by DDD) as a factor influencing AMR. As a result, a correlation was proved for *E. coli* between AMC (protected penicillins, third-generation cephalosporins and protected cephalosporins) and AMR. The aim of the present study was to construct a national bank of mathematical models predicting change in the level of antimicrobial resistance on the basis of data about consumption of antibiotics and the current level of AMR.

## Materials and methods

### Information about antibiotic consumption

From the IQVIA database (Russia and the Commonwealth of Independent States – CIS) containing information about the sale of medicines we obtained data about the consumption of antibiotics for systemic use (code J01 under the ATC classification system) in the period from 2008 to 2022 for 82 regions of the Russian Federation. The database held information about 15 groups of antimicrobials, the level of consumption for each of them at the outpatient and inpatient stages of treatment, consumption of the antibiotics in DDDs. When we processed the information provided, each of the antibiotics was assigned to a particular sub-class in accordance with Anatomical Therapeutic Chemical (ATC) classification (ATC/DDD Index, 2024).

### Information about AMR

Information about AMR for clinically significant pathogens in the period 2013–22 was drawn from the database of the resource AMRmap.ru–a system for interactively monitoring AMR at the national level. The AMRmap.ru database consists of data from prospective multi-centre studies into AMR carried out by the Institute of Antimicrobial Chemotherapy (IAC) of Smolensk State Medical University of the Ministry of Health of the Russian Federation in collaboration with IACMAC – the Interregional Association for Clinical Microbiology and Antimicrobial Chemotherapy (Kuzmenkov et al., 2021). The data utilized contain information about clinically significant isolates obtained from over 100 participating centres. All the isolates had been identified down to types, and the activity of antimicrobials was assessed using a reference broth microdilution technique (ISO). Since the data were presented in the form of minimum inhibitory concentration (MIC), the clinical category of susceptibility was established by unified criteria (in accordance with EUCAST) for the entire data pool, including historical data, which provided for the comparability of the established susceptibility categories (European Committee on Antimicrobial Susceptibility Testing, 2022). The final set of data contained information about the type of pathogen; the region of Russia where the pathogen was isolated; the year when the material was taken; the type of infection (community-acquired or nosocomial); the results of determining susceptibility to antimicrobials. Model pairs were examined separately for the class of antibiotic and the microorganism. The regions with the least number of observations for each microorganism-antimicrobial pair were identified individually and excluded from the study.

### Data preparation

The information obtained from various sources was reduced to a unified form: among other things, a matching dictionary was compiled for the names of the regions of the Russian Federation, and also for the names of the drugs active ingredients. Information about the volume of antimicrobial consumption in topical dosage forms was not taken into account. Antimicrobials with insignificant usage in hospitals or retail pharmacies were excluded from the analysis. The analysis included medications accounting for 95% of the total volume of consumption, sorted by total DDDs in decreasing order.

The following factors were considered in the analysis:

- Year of consumption/year of conducting tests for AMR (taking account of the time component);
- Type of infection: community-acquired or nosocomial (to give the ability to take account of differences in the dynamics of antibiotic consumption for the hospital and community sectors);
- The type of antimicrobial consumption base (hospital or retail pharmacy);
- The total number of DDDs for various antibiotics over a year within each of the bases for each region of the Russian Federation (RF);
- The results of determining the susceptibility of each microorganism to each antimicrobial for each region of the RF;

By way of a supplementary factor for modelling, we examined the moving aggregate AMR by region and antibiotic-microorganism (separately for community-acquired and nosocomial infection) with a one-year time lag, with a sliding window of three to 10 years. To minimize the influence of random and inexplicable fluctuations caused by unaccounted factors, we used a moving average. Such an approach intended to take into account the accumulated influence of past AMR values, smooth the data, and allow the identification of long-term trends, despite the noise and possible unaccounted variables. In all subsequent analyses, only moving average AMR by region and antibiotic-microorganism (separately for community-acquired and nosocomial infection) were used as input features.

Consumption factors were analysed in the form of DDDs at both the outpatient and inpatient stages, separately for each database (hospital or retail pharmacy). The consumption of each class of antimicrobial was included in the analysis as a separate feature. Additional calculated features (moving averages) were taken into account reflecting consumption over previous moving periods of three to 5 years, taking the regional profile into consideration. The addition of moving averages over preceding periods was necessary in order to calculate the cumulative effect of the consumption of various antibiotics.

We used principle component analysis to transform the features associated with consumption (antimicrobials DDDs data and their moving averages over previous periods – 40 factors in all) into five main components, which allowed us to preserve around 90% of the information (explained variance). This was done to reduce noise and correlation between factors, since the consumption dynamics for some classes of antibiotics demonstrate a high level of cross-correlation. In all subsequent analyses, only these first five principal components were used as input features.

### The model pair

In view of the complexity and diversity of the processes that lie behind the formation and spread of AMR, within the framework of the present study we propose the concept of a “model pair” – a combination of one microorganism and one antimicrobial with a forecast being produced for the proportion of isolates of the given microorganism resistant to the given antimicrobial. For a selected model pair we constructed various machine-learning models using the data pool that had been assembled. The input features for the model pair include the following elements: historical moving averages of AMR for the specific pair; the type of infection (community-acquired/nosocomial); the five principal components derived from antibiotic consumption data; regional identifier; and time (year). For a given model pair in a specific region and year, the model’s output is the predicted proportion of isolates classified as resistant to the antimicrobial agent. Table 1 provides a detailed description of the input features and the target variable used in the machine learning models for predicting antimicrobial resistance. For the final aggregated annual assessment, the model-predicted values for each individual region were combined on a per-year basis using a weighted average. The weighting factor for each region reflected the number of isolates with available susceptibility data for the specific antibiotic in that region for the corresponding year.

**TABLE 1:** Description of input features and the target variable for the model pair.

Variable	Type	Measurement/Encoding	Description
Target variable (aggregated)	Continuous	The proportion of resistant isolates	Predicted AMR level: The proportion of isolates classified as resistant within a given region and year. For the final aggregated annual assessment, the model-predicted values for each individual region were combined on a per-year basis using a weighted average. The weighting factor for each region reflected the number of isolates with available susceptibility data for the specific antibiotic in that region for the corresponding year
Historical AMR for the specific “model pair” (moving avg.)	Continuous	%	Smoothed historical resistance level (3–10 years window) with a 1-year lag
Type of infection	Categorical	One-hot: [0,1] = community, [1,0] = nosocomial	Distinguishes between community-acquired and hospital-acquired infection settings; furthermore, for consumption data, the encoding [0,1] corresponds to the retail pharmacy sector (outpatient), while [1,0] corresponds to the hospital sector (inpatient)
Antibiotic consumption (PCA components)	Continuous	Dimensionless principal components (PC1–PC5)	Transformed consumption data (in DDDs) for features associated with consumption (antimicrobials DDDs data and their moving averages over previous periods – 40 factors in all) across hospital or retail pharmacy sectors
Region	Categorical	One-hot encoding	Account for regional characteristics and variations
Year	Continuous	Year of observation (integer)	Accounts for overall time trend

### Machine learning approach

In our mathematical modelling of the AMR dynamics we employed the following methods:

- A gradient boosting model (Light Gradient Boosting Machine – LightGBM) (Ke et al., 2017). This is a highly effective gradient boosting model that uses algorithms based on decision trees. Boosting is a method of machine learning that combines several simple models (usually decision trees) to create a single powerful model. In the boosting process, each fresh model learns from the errors of its predecessors, making it possible to gradually improve the quality of predictions and reduce errors. LightGBM optimizes the learning process, employing methods that reduce the number of calculations, such as “leaf-wise” growth and histogram-based splitting that enables LightGBM to achieve outstanding productivity and scalability when working with large volumes of data and high dimensionality of features. A limitation of the method is that modelling using LightGBM may reduce forecasting precision when using data that differ strongly from those used in training. Furthermore, like other gradient boosting methods, LightGBM can be inclined to overfitting, particularly when working with small or noisy sets of data.
- Logistic regression (Cox, 1958). This widely used statistical method of machine learning is particularly effective when working with binary problems. The method is used to model the probability of an object belonging to one of two classes. The model evaluates probability using a logistic function (sigmoid function) that transforms the linear combination of features into a value between 0 and 1. The advantage of logistic regression lies in its high interpretability and simplicity of implementation. It works well on linearly separable data and is resistant to overfitting, especially when regularization is used. Logistic regression can be ineffective when working with non-linearly separable data and high dimensional data. In such cases, the model may present low-precision predictions. Further more, logistic regression assumes the absence of strong multicollinearity between features, which may require preliminary processing of the data.
- The random forest approach (Breiman, 2001). This is a powerful machine learning model that employs an ensemble approach to increase the precision of predictions. It is a method that combines multiple decision trees to produce a single strong model. In the training process, each tree is constructed on a random subsample of data and features, which makes it possible to reduce correlation between trees and improve the overall productiveness of the model. The random forest has high resistance to overfitting and can process large volumes of data with many features efficiently.
- The support vector method with a Gaussian kernel (Support Vector Machine, SVM) (Cortes and Vapnik, 1995). This powerful method of machine learning, used for classification and regression, is particularly effective when working with non-linearly separable data. It makes it possible to perform a search for an optimal hyperplane for dividing the data into classes. The Gaussian kernel (also known as the radial basis function, RBF) enables the SVM to model the complex, non-linear boundaries between classes, transforming the data to a higher dimensionality. The SVM with a Gaussian kernel has a high capacity for generalization and can efficiently process complex, non-linear data. By employing an RBF kernel, the model is capable of creating flexible separation boundaries, permitting the achievement of high precision in predictions.
- The support vector method with a linear kernel (Cortes and Vapnik, 1995). This powerful method of machine learning, used for classification and regression, is particularly effective when working with linearly separable data. The linear kernel in the SVM is used to create linear boundaries between classes, which makes it a straightforward and effective tool for tasks where the classes can be divided by a linear hyperplane. The SVM with a linear kernel has a high computational efficiency and can process large volumes of data rapidly. The linear kernel is particularly useful when working on tasks where the data are linearly separable and it provides the model with a high level of interpretability.

### Calibration and validation of the models

We divided the data contained in the database into training and validation sets. Cross-calibration of the hyperparameters for each model was carried out on the training set, after which the chosen metric was evaluated on the validation set. This process was repeated 100 times using random subsamples of the original data to construct an interval estimate for the metric.

The basis parameters of the LightGBM, such as the number of trees, speed of learning, maximum depth and number of leaves, were optimized to achieve a balance between the complexity of the model and its capacity to generalize data. The parameters of a subset of features and samples were also used to reduce correlation between trees and increase the model’s stability. Regularization methods, such as limiting the depth of the trees and adding noise in the data, were also employed to improve the overall performance of the LightGBM model.

To tune the random forest model we tested various values for the number of trees so as to strike a balance between precision and time taken in learning. The maximum depth of trees was restricted to prevent overfitting. The portion of features used to tune each tree was also optimized, which reduced correlation between trees and enhanced the model’s capacity to generalize data. The parameters determining the minimum quantity of samples for the separation of nodes and the minimum quantity of samples in the leaves were optimized to achieve the best balance between overfitting and underfitting.

To tune the logistic regression model, we selected initial parameters such as the optimization method and the inclusion of the intercept in the equation. During the calibration process, the parameters related to regularization were optimized to find the best level of penalty for the complexity of the model. We also examined different values for the maximum number of iterations to ensure a stable and precise solution. Those steps allowed us to improve the model’s precision and its capacity to generalize data.

To tune the support vector model, we selected initial parameters such as the regularization level, convergence accuracy and probability consideration. Class balance was also taken into account to improve precision. Support vector machines (SVMs) usually have good generalizability and are resistant to overfitting. In our case, SVM calibration was not performed due to the time consumption and computational complexity of the process, and also because the initial parameters of the model already provided satisfactory results on the validation set.

For the final model, constructed on the basis of averaged hyperparameters for all calibrations, we carried out an assessment of the importance of factors and a simulation analysis. Assessing the importance of factors is an important step in analysing machine learning models, as it makes it possible to understand which features have the greatest impact on the model’s predictions.

In the LightGBM model, the importance of features was determined by summing the reduction in the loss function for all partitions in which the feature was involved, and averaging this value over the number of such splits. This makes it possible to assess how much each feature contributes to improving the quality of the model.

In the random forest, the importance of the features was determined by the average decrease in Gini impurity when a particular feature is used to split nodes.

In logistic regression, the importance of the features was determined using the influence of each on the probability of belonging to a particular class.

During the simulation analysis, the consumption of the class of antimicrobial under consideration was successively changed by a fixed delta over the entire series of consumption data. This allowed us to study how changes in the level of consumption affect the predicted values and the behaviour of the model as a whole. Thus, for each class of antimicrobial, AMR was evaluated in accordance with different levels of consumption, which made it possible to determine the model’s sensitivity to changes in the data and to identify potential risks or opportunities associated with fluctuations in consumption. The results of the analysis can be used to plan resources more precisely and make strategic decisions regarding the management of medicines.

### Forecasting for future periods

We forecast AMR for future periods, determining the confidence interval by the bootstrap method. The values were predicted step by step, taking account of previous data for each new forecast, among other things to update the moving averages. The AMR forecast was based on both expected and optimal levels of antimicrobial consumption, providing a more precise understanding of possible resistance change scenarios. In this way, we obtained two forecasts – an optimal one and a realistic one.

The optimal forecast selected levels of antimicrobial consumption that led to the minimum possible value for AMR. This forecast made it possible to determine the very best conditions (in this case the structure and level of antimicrobial consumption), under which the AMR level would reach its extremum (minimal value).

The selection of optimal levels of antimicrobial consumption was performed using COBYLA method (Constrained Optimization BY Linear Approximations) (Powell, 2007). That method is intended for solving non-linear optimization problems with constraints, using linear approximations to seek optimal solutions. COBYLA allowed us to calculate optimal levels of antimicrobial consumption that minimize the growth in resistance taking account of given restraints (it was assumed that consumption cannot have a negative value or be more than double the previous year’s figure). The realistic forecast on the basis of expected levels of antimicrobial consumption was based on ETS (exponential trend smoothing) model (Hyndman and Athanasopoulos, 2018). Consumption data by year were aggregated for particular antimicrobials in a cross-section of hospital and outpatient stages of medical care. Outliers were removed using the interquartile range (IQR) method, while missing values were filled in by linear interpolation.

After that, the calculated total consumption forecast for each class of antimicrobial was distributed among regions in accordance with the observes shares of each region’s contribution to total consumption. These regional data were used as input to the model to perform further AMR forecasting.

### Automated data preparation

The processes of data preparation and preprocessing, as well as model training were implemented as executable Python scripts to provide for the reproducibility of the research, as well as to create a continuous data processing pipeline with the aim of automating the subsequent creation and deployment of machine learning models.

### Delivery of the models to end users

In order to provide access to the developed models and visualization of the data on antimicrobial consumption, an open online platform AMCmodel.ru was created with a collection of constantly updated models and visualization tools.

## Results

The pair selected for study was *E. coli* and cefotaxime. This choice can be explained by the fact that *E. coli* is the microorganism most frequently isolated in microbiological studies in the Russian Federation, which ensured a sufficient volume of data for analysis, while the study of this particular antibiotic-pathogen pairing had potential for the optimization of therapy within the framework of national recommendations. Five different models were constructed for the *E. coli*-cefotaxime pair. During the modelling process, the entire dataset was divided up into training (70%) and validation (30%) sets. Hyperparameter optimization was performed for each model using cross-validation. As a result, the LightGBM model showed the best results, demonstrating high precision and robustness to data noise (Table 2).

**TABLE 2:** Evaluation of the precision and recall of various types of models on test and training sets.

Model	Type of data	Classification accuracy			Precision			Recall		
		Mean	std dev	CI	Mean	std dev	CI	Mean	std dev	CI
LightGBM	Test	67.1%	0.8%	(0.669, 0.672)	65.2%	1.2%	(0.65, 0.655)	69.3%	2.3%	(0.689, 0.698)
LightGBM	Training	67.9%	0.8%	(0.677, 0.68)	66.3%	1.1%	(0.66, 0.665)	69.4%	2.0%	(0.69, 0.698)
Random forest	Test	67.0%	0.7%	(0.669, 0.671)	65.0%	1.2%	(0.647, 0.652)	69.9%	1.7%	(0.695, 0.702)
Random forest	Training	67.8%	0.7%	(0.677, 0.68)	66.0%	1.1%	(0.658, 0.662)	70.2%	1.6%	(0.698, 0.705)
Logistic regression	Test	66.3%	0.6%	(0.662, 0.664)	65.3%	1.0%	(0.651, 0.655)	65.8%	1.8%	(0.654, 0.661)
Logistic regression	Training	66.7%	0.6%	(0.666, 0.668)	65.7%	0.8%	(0.655, 0.659)	66.2%	1.4%	(0.659, 0.664)
SVM, RBF kernel	Test	66.4%	0.6%	(0.663, 0.665)	64.2%	0.9%	(0.64, 0.644)	70.3%	1.3%	(0.7, 0.705)
SVM, RBF kernel	Training	66.7%	0.6%	(0.666, 0.668)	64.4%	0.8%	(0.643, 0.646)	70.4%	1.3%	(0.702, 0.707)
SVM, linear kernel	Test	65.8%	0.6%	(0.657, 0.659)	65.0%	0.8%	(0.648, 0.651)	64.4%	0.9%	(0.642, 0.645)
SVM, linear kernel	Training	65.8%	0.6%	(0.656, 0.659)	65.0%	0.8%	(0.649, 0.652)	64.4%	0.9%	(0.642, 0.645)

LightGBM, Light Gradient Boosting Machine; SVM, Support Vector Machine (with Gaussian/RBF, or linear kernel); CI, 95% confidence interval.

**FIGURE 1:**
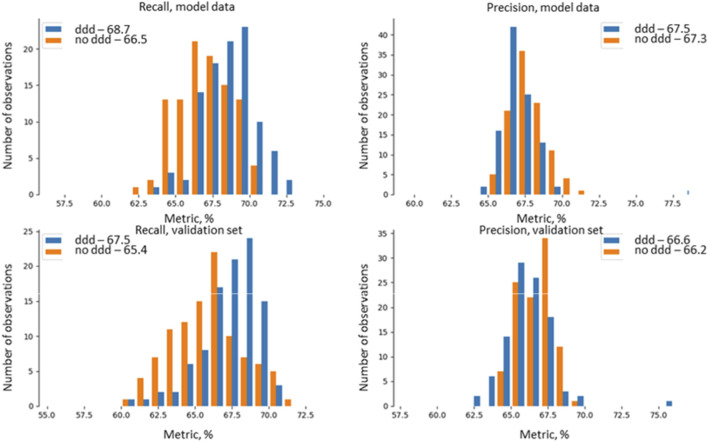
Interval assessments of model quality metrics (precision and recall) for the training and validation data sets for the *E. coli*–cefotaxime pairing.

Figure 1 shows interval assessments of model quality metrics (precision and recall) for the training and validation data sets. The distributions are roughly the same for both of them. For example, recall on the training data reaches 68.7%, with values ranging from 62.5% to 72.5%. On the validation data, the values ranged from 60% to 71%, with a maximum of 67.5%. Similar results were obtained for precision: on the model data the most frequently occurring figure was 67.5% (with a range from 65% to 71%), and 66% for the validation set. The precision and recall values on the training and validation set are close (the difference is <5%), which attests to an absence of overfitting.

The small reduction in values on the validation data set (67.5% → 66.6%) is an anticipated phenomenon, indicative of the model’s good generalization ability. At the same time, the spread of the values for the metrics indicates the sensitivity of model to variations in the data.

The importance of features in the LightGBM and Random Forest models was assessed based on the contribution of a particular feature to the reduction of the loss function.

**FIGURE 2:**
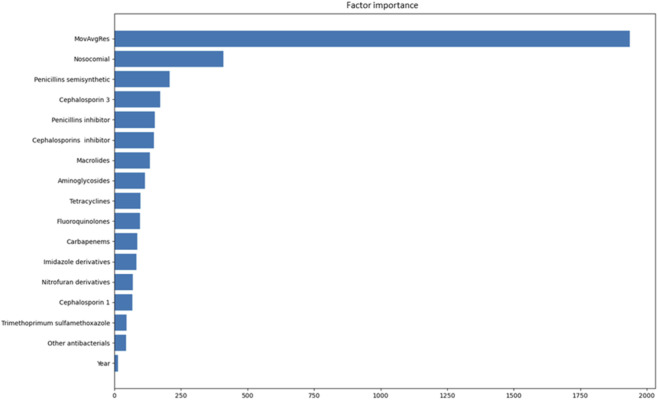
Importance of the factors (gain metric, LightGBM model).

As can be seen from Figure 2, the greatest contribution was made by the moving average of resistance to the antimicrobial over the preceding periods, as well as the type of infection (community-acquired or nosocomial).

**FIGURE 3:**
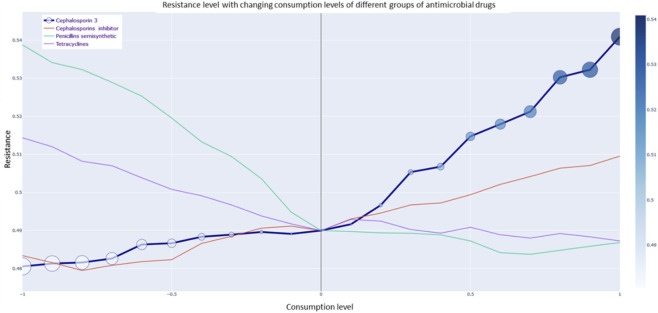
Sensitivity analysis of results with changes in the levels of antibiotic consumption.

To assess the sensitivity of the model we carried out a simulation analysis of the model behaviour with changes in the levels of antibiotic consumption (Figure 3).

**FIGURE 4:**
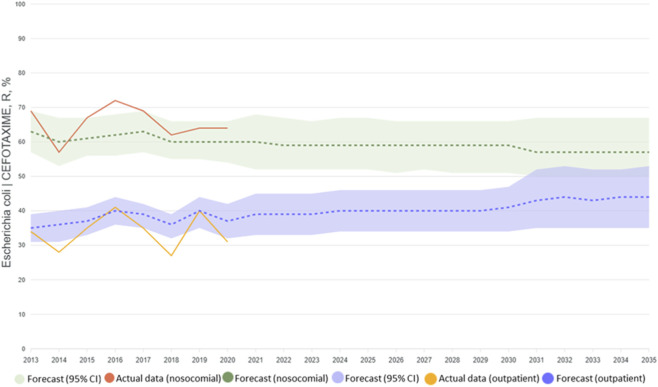
The dynamics of AMR – results of modelling according to the optimal consumption scenario.

For the *E. coli*–cefotaxime model pair two types of 10-year forecasts were constructed separately for the community-acquired and nosocomial segments: a scenario with an optimal level of consumption and a realistic one. In the case of the optimal scenario, the levels of antimicrobial consumption were selected by the COBYLA method to minimize the predicted level of AMR (Powell, 2007). In doing so, we took into account that consumption could not be negative or exceed a twofold increase in the value relative to the previous year (Figure 3). The results obtained using the LightGBM model for the optimal scenario present a forecast reduction in resistance of 15%–20% when consumption structure is optimized (Figure 4).

**FIGURE 5:**
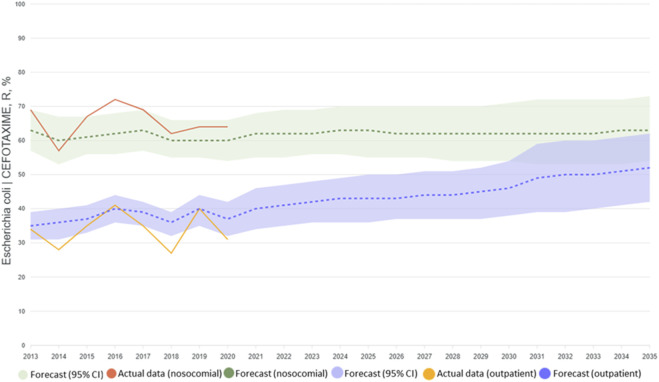
The dynamics of AMR – results of modelling according to the realistic consumption scenario.

The realistic forecast was based on expected levels of consumption, calculated using the ETS (exponential smoothing) model. An example of the estimate of future consumption is shown in Figure 5.

As can be seen from Figure 5, on the basis of the realistic scenario the LightGBM model shows a growth in resistance of 5%–10% if present tendencies continue. It is notable that when current levels of *E. coli* isolates’ resistance to cefotaxime and of consumption of the antibiotic persist, the forecast predicts a significant grown in resistance among community-acquired isolates. Such a forecast probably reflects the existence of a certain potential for the development of an even greater level of resistance in non-hospital settings.

The model constructed on the basis of LightGBM makes it possible to predict the dynamics of AMR with a high degree of accuracy and can be used to develop strategies for the management of antimicrobial therapy. The realistic forecast warns about the risk of a growth in resistance if current practice is retained.

In order to generalize the concept of the “model pair” and provide access to the models created, as well as to data on antimicrobial consumption, the AMCmodel.ru (AntiMicrobial Consumption model) was developed, where users can access interactive infographics on antimicrobial consumption, as well as an interface to interact with the models that have been created to generate a forecast of resistance for a given model pair. This portal serves as a universal repository (bank) of models for their efficient fine-tuning and delivery to the end user in the form of an interactive interface.

Further research will be aimed at expanding the bank of models for other pathogen–antibiotic pairs and integration of data from international databases for a global analysis of AMR.

## Discussion

Given the growing threat of antimicrobial resistance (AMR), forecasting models have a key role to play in the development and implementation of effective strategies for the use of antibiotics. These models, based on state-of-the-art methods of machine learning and statistical analysis, make it possible not only to predict the development of resistance, but also to correct the structure of the consumption of antibiotics, adapting their usage to regional characteristics and current epidemiological trends (Cesaro et al., 2025). Forecasting AMR is a complex task, requiring the integration of various types of data and the use of advanced methods of machine learning. In recent years, considerable attention has been devoted to modelling the dynamics of resistance to antibiotics using algorithms such as neural networks, Light Gradient Boosting Machine (LightGBM) and Extreme Gradient Boosting (XGBoost). However, the choice of algorithm depends not only on its capacity for precise forecasting, but also on the characteristics of the data. As a rule, data on AMR is characterized by class imbalance that makes training models more difficult. In the course of the present study, we developed a system for the predictive modelling of AMR dynamics based on machine learning. The results obtained demonstrate the potential that mathematical modelling has for forecasting changes in the level of resistance and optimizing strategies for the usage of antibiotics.

The results bear out the hypothesis regarding a direct correlation between the consumption of antibiotics and the development of AMR that was advanced by the WHO and confirmed in previous works, including Russian-based studies (Arepyeva et al., 2017; Lv and Wang, 2024). The use of machine learning makes it possible to take account of non-linear correlations between variables, which increases the precision of the forecast compared to classical regression models (Ardila et al., 2025). It should be pointed out that the approach to the construction of machine-learning models described in the present study expands existing methods, bringing together data on antibiotic consumption (in DDD units), local resistance and factors influencing the epidemiological situation (type of infection, regional peculiarities). Additionally, the use of time series for the analysis of AMR dynamics allowed us to take account of lag effects and seasonal fluctuations. For example, data from the Veterans Health Administration (VHA) have shown that long-term trends in antibiotic usage show considerable variation (with a median up to 0.8), while seasonality is not a dominant factor (Li et al., 2025). These conclusions accord with the results of other studies where machine learning has been successfully employed to forecast AMR on the basis of data on the consumption of antimicrobials (Tsuzuki et al., 2025).

From the results of the study that we carried out, the most successful model proved to be LightGBM, which displayed high resistance to overfitting and forecasting precision (67.5% on the training set and 66.6% on the validation set). That accords with the particular characteristics of an algorithm that makes it possible to efficiently process high-dimensional and noisy data (Sakagianni et al., 2023). Feature importance analysis revealed that the moving average proportion of resistant microorganisms, the type of infection (community-acquired or nosocomial), as well as the historical level of consumption of antimicrobials, are key factors influencing resistance.

The forecasting scenarios examined showed that optimization of the consumption structure for antibiotics can reduce the level of AMR to cefotaxime by 15%–20% over 10 years. That is of strong significance for the development of national programmes of antimicrobial therapy management, especially in the context of WHO targets under the AWaRe classification, where antimicrobials in the Access group are supposed to account for 60% of consumption (Mudenda et al., 2023). However, the realistic scenario warns us of a 5%–10% growth in resistance, if current trends continue, especially among non-hospital isolates of *E. coli*. Meanwhile, for nosocomial isolates the predicted level of resistance remains high and practically unchanged. That points to a need for stronger control over the use of antibiotics in outpatient therapy, where considerable potential for a reduction in resistance can be observed. These facts confirm, among other things, the hypothesis about the cumulative effect of the use of antimicrobials, which has previously been noted in works on modelling infectious diseases (Mudenda et al., 2023).

The portal AMCmodel.ru created in the course of the present study provides access to interactive models and data about the consumption of antibiotics, which opens up possibilities for their prompt further training and delivery to the end user in the form of an interactive interface. Further studies will be directed towards expanding the bank of models for other pathogen–antibiotic pairs and integration of data from international databases for a global analysis of AMR. The use of AI methods such as convolutional neural networks (CNNs) and recurrent neural networks (RNNs) will permit analysis of complex patterns of data. That will create a foundation for more precise and individualized forecasts of AMR (Arnold et al., 2025).

The constraints of the present study deserve special consideration: the limited availability of regional data on consumption and resistance, especially for the early years, may have affected the precision of the model. Furthermore, the models developed were only tested on the model pair *E. coli*–cefotaxime. To increase universality, the bank of models needs to be expanded to include other pathogens and antimicrobials. Additionally, the model does not take into account such variables as changes in the actual practice of antimicrobial therapy resulting from improved clinical recommendations.

The model does not reckon with the possibility of fresh mechanisms of resistance appearing that might fundamentally alter the character of relations in the microbe–antibiotic model pairs. One example of such a catastrophic event has been the emergence and spread of carbapenemase enzymes, which undermined the effectiveness of a highly important class of antibiotics – the carbapenems. Given the fact that historical levels of resistance and antimicrobial consumption are key factors influencing resistance, it is not clear whether the model will be able to predict the spread of resistance to new antibiotics that have only recently entered healthcare, such as combination drugs based on beta-lactams and beta-lactamase inhibitors from the two groups of diazabicyclooctanes and boronic acid derivatives.

Hence, to further improve AMR forecasting systems there is a necessity to concentrate on expanding the capabilities of machine learning and the integration of data from different sources. Particular attention should be devoted to the continuous creation and updating of models, taking account of regional and local peculiarities, which will make it possible to minimize the risks associated with AMR.

Present-day approaches to the forecasting and management of AMR are demonstrating significant progress due to the employment of machine learning methods, deep analysis of the data and integration of databases. The results obtained confirm that machine learning is a powerful tool for predicting AMR and developing strategies for managing antimicrobials. The implementation of recommendations based on the proposed models can contribute to the achievement of the WHO’s targets on the reduction of mortality from antimicrobial resistance by 2050 (Elalouf et al., 2025). Neverthelss, the successful implementation of these models requires taking account of regional peculiarities, standardization of data and adaptation to clinical conditions.

## Data Availability

The original contributions presented in the study are included in the article, further inquiries can be directed to the corresponding author.
